# Combining expert and crowd-sourced training data to map urban form and functions for the continental US

**DOI:** 10.1038/s41597-020-00605-z

**Published:** 2020-08-11

**Authors:** Matthias Demuzere, Steve Hankey, Gerald Mills, Wenwen Zhang, Tianjun Lu, Benjamin Bechtel

**Affiliations:** 1 Department of Geography, Ruhr-University Bochum, Bochum, Germany; 2 School of Public and International Affairs, Virginia Polytechnic Institute and State University, Blacksburg, USA; 3 School of Geography, University College Dublin, Dublin, Ireland

**Keywords:** Environmental impact, Environmental health

## Abstract

Although continental urban areas are relatively small, they are major drivers of environmental change at local, regional and global scales. Moreover, they are especially vulnerable to these changes owing to the concentration of population and their exposure to a range of hydro-meteorological hazards, emphasizing the need for spatially detailed information on urbanized landscapes. These data need to be consistent in content and scale and provide a holistic description of urban layouts to address different user needs. Here, we map the continental United States into Local Climate Zone (LCZ) types at a 100 m spatial resolution using expert and crowd-sourced information. There are 10 urban LCZ types, each associated with a set of relevant variables such that the map represents a valuable database of urban properties. These data are benchmarked against continental-wide existing and novel geographic databases on urban form. We anticipate the dataset provided here will be useful for researchers and practitioners to assess how the configuration, size, and shape of cities impact the important human and environmental outcomes.

## Abstract (summary)

**Table:** 

Measurement(s)	local climate zones
Technology Type(s)	digital curation
Factor Type(s)	building footprint • building height • impervious surface area • Sky view factor • anthropogenic heat flux • population
Sample Characteristic - Environment	anthropogenic environment • city
Sample Characteristic - Location	contiguous United States of America

Machine-accessible metadata file describing the reported data: 10.6084/m9.figshare.12599966

## Background & Summary

There is a scientific consensus on the need for spatially detailed information on urban landscapes at a global scale to support a range of environmental services^1^. The consensus has emerged as the simulation and forecasting capabilities of models have improved radically over the last decade and the demand for reliable urban-scale information that can inform policies has increased^2^. The latter is an acknowledgment of cities as places of: intense resource consumption and waste generation and; foci of population and infrastructure that are exposed to multiple hazards of natural and anthropogenic origin. A number of projects have mapped the global urban extent at finer and finer detail^3–6^ but most do not account for the spatial variation within cities, that is, the urban layout as a consequence of historic urbanization patterns that reflect local terrain, culture, economy, etc. These data are needed as part of a basic infrastructure to support a host of studies on historical urbanization processes^7–9^, transportation behavior^10,11^, exposure to environmental hazards^12–17^, energy demand^18^, climate mitigation solutions^19^ and human health^20^, as examples. Moreover, the data are needed to support planning decisions that can address aspects of urban form (e.g. green fraction) and function (e.g. transportation networks) to mitigate the urban impact^1,21–23^. While these data can be assembled for data-rich cities, the challenge is to acquire sufficient information at very large scales using a consistent methodology. While there are established geographical databases on natural land-cover that have been derived using consistent methodologies, until recently there has been no equivalent for urban land-cover types. Since 2000 there have been a number of initiatives at mapping population and building land-covers at global scales using earth observation data, census and building footprint information^24–26^. However, these need to be complemented by a landscape approach that can distinguish urban surfaces on a holistic basis, which accounts for the typical combination of micro-scale land-covers and associated physical properties.

The Local Climate Zone (LCZ) typology is a good candidate for such classification scheme^27^. Each of the 10 urban types are linked to a series of relevant variables that describe the physical characteristics of that type. In an urban environment, each LCZ refers to a neighborhood (sized about 1 km^2^) that is relatively homogeneous in terms of urban layout, that is the spatial coherence of the attributes (the types of buildings, green space, impermeable fraction, etc.) that distinguish one area from another. The product is distinguished from other urban typologies (e.g.^26,28,29^) which focus on individual aspects of urban cover (e.g. residential, commercial and industrial) without providing ancillary information. Critically, the LCZ scheme can be employed to examine the nature of urbanization and its likely environmental impact^30^. The World Urban Database Access Portal Tools (WUDAPT) project has adopted the LCZ scheme as a basic description of urban land-cover and has developed a process for automatically classifying urban areas globally into types using satellite imagery and training areas^31^. Moreover, experiments have shown that training areas derived for one city can be used to classify other urban areas with certain caveats. Demuzere *et al*.^32^ demonstrated that this insight could be harnessed within the computation environment of Google’s Earth Engine^33^ to access a suite of remote sensing data and effectively map LCZ types at the scale of Europe^34^.

In this paper, expert derived training data (the original approach developed by WUDAPT) are combined with crowd-sourced training data from Amazon Mechanical Turk (MTurk, https://www.mturk.com/https://www.mturk.com/) to create a gridded, high-resolution, Local Climate Zone map for the continental United States (CONUS). This resulting map is evaluated using a variety of available geographical databases that provide information on individual aspects of the urban landscape, such as impermeability, building footprints and heights. These data will be of value to a variety of users because of the consistency of methodology and scale. For example, continental-scale studies of environmental hazards are often limited by inconsistent data of varying quality to describe land use and urban form^35–38^. Similarly, research that assesses how the built environment impacts travel behavior (e.g., alternative transportation)^39–41^, physical activity^42,43^, and mental health^44^ is often limited to a single urban area or suffers from crude or inconsistent measures across large geographies. We anticipate the dataset provided here will be useful for researchers and practitioners to assess how the configuration, size, and shape of cities impact these important human and environmental outcomes. More generally, our approach demonstrates the potential for integrating crowd-sourced data into LCZ model-building for the purpose of developing global LCZ maps.

## Methods

### Concept of local climate zones

**Fig. 1:**
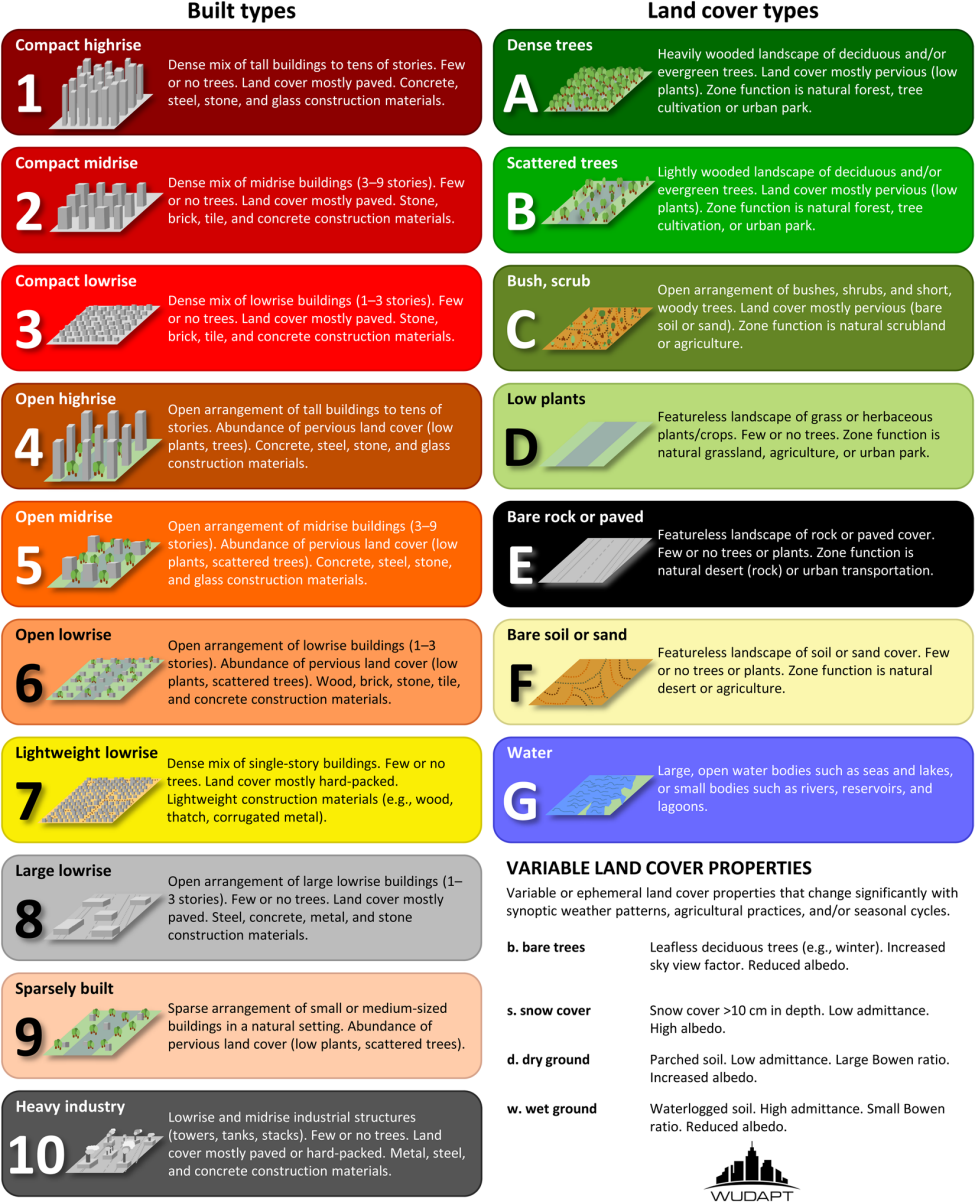
Urban (1–10) and natural (A–G) Local Climate Zone definitions (adapted from Table 2 in Stewart and Oke^27^, default LCZ colors according to Bechtel *et al*.^31^).

LCZ typology has been adopted as a baseline description of global urban areas into recognisable types that are formally defined as ‘regions of uniform surface cover, structure, material, and human activity that span hundreds of meters to several kilometres in horizontal scale’, exclude ‘class names and definitions that are culture or region specific’, and are characterized by ‘a characteristic screen-height temperature regime that is most apparent over dry surfaces, on calm, clear nights, and in areas of simple relief’^27^. Seventeen LCZ types exist, 10 of which are considered ‘urban’ (Fig. 1), and all are associated with characteristic urban canopy parameters (UCP, Table 1). In the current study, two ‘urban’ LCZ classes are not considered: LCZ 7 (lightweight lowrise) referring to informal settlements hardly present in CONUS, and LCZ 9 (sparsely built) characterised by a high abundance of natural land-cover which thus behaves thermally as a natural land-cover.

**Table 1:** Urban canopy parameter data associated with LCZ types, based on Stewart and Oke^27^ and adapted from Demuzere *et al*.^32^.

LCZ	*λ*_*B*_	*λ*_*I*_	*λ*_*V*_	H	SVF	AHF	ISA
1. Compact highrise	40–60	40–60	<10	>25	0.2–0.4	50–300	>80
2. Compact midrise	40–70	30–50	<20	10–25	0.3–0.6	<75	>70
3. Compact lowrise	40–70	20–50	<30	3–10	0.2–0.6	<75	>60
4. Open highrise	20–40	30–40	30–40	>25	0.5–0.7	<50	50–80
5. Open midrise	20–40	30–50	20–40	10–25	0.5–0.8	<25	50–80
6. Open lowrise	20–40	20–50	30–60	3–10	0.6–0.9	<25	40-90
7. Lightweight lowrise	60–90	<20	<30	2–4	0.2–0.5	<35	>60
8. Large lowrise	30–50	40–50	<20	3–10	>0.7	<50	>70
9. Sparsely built	10–20	<20	60–80	3–10	>0.8	<10	10-40
10. Heavy industry	20–30	20–40	40–50	5–15	0.6–0.9	>300	>40
A. Dense trees	<10	<10	>90	3–30	<0.4	0	<20
B. Scattered trees	<10	<10	>90	3–15	0.5–0.8	0	<20
C. Bush, scrub	<10	<10	>90	<2	0.7–0.9	0	<20
D. Low plants	<10	<10	>90	<1	>0.9	0	<20
E. Bare rock or paved	<10	>90	<10	<0.25	>0.9	0	>90
F. Bare soil or sand	<10	<10	>90	<0.25	>0.9	0	<20
G. Water	<10	<10	>90	—	>0.9	0	<20

Columns represent the building footprints (*λ*_*B*_ [%], ratio of building plan area to total plan area), impervious (*λ*_*I*_ [%], ratio of impervious plan area (paved, rock) to total plan area) and vegetated (*λ*_*V*_ [%], ratio of pervious plan area (bare soil, vegetation, water) to total plan area) land-covers, mean height of roughness elements (H [m], geometric average of building heights (LCZs 1–10) and tree/plant heights), sky view factor (SVF) and anthropogenic heat flux (AHF [W m^−2^]). The last column presents the total impervious surface area (ISA [%]), calculated as the sum of the outer ranges of *λ*_*B*_ and *λ*_*I*_.

An automated (offline) LCZ mapping procedure was suggested by Bechtel *et al*.^31^, and adopted by the WUDAPT project to create consistent LCZ maps of global cities. To facilitate the expansion of the coverage of LCZ maps, Demuzere *et al*.^32,34^ introduced the transferability concept of labeled Training Areas (TAs)^32^ and the use of Google’s Earth Engine cloud computing environment^33^, which allows for up-scaling the default WUDAPT approach to the continental scale (e.g. Europe^34^). In this approach, the three key operations from the original WUDAPT protocol remain unchanged: (1) the preprocessing of earth observation data as input features for the random classifier, (2) the digitization and preprocessing of appropriate training areas, and (3) the application of the classification algorithm and the accuracy assessment^45^. These steps are described in more detail below.

### Input data

#### Earth observation data

The default WUDAPT workflow typically uses a number of single Landsat 8 (L8) tiles as input to the random forest classifier^31^. Here we adopt the approach of Demuzere *et al*.^34^, by using 41 input features from a variety of sensors and time periods. L8 mean composites (2016–2018) are made for the full year and the summer/winter half-year, and include the blue (B2), green (B3), red (B4), near-infrared (B5), shortwave infrared (B6 and B7) and thermal infrared (B10 and B11) bands. In addition, a number of spectral indices are derived (as composites covering the period 2016–2018): the minimum and maximum Normalized Difference Vegetation Index (NDVI), the Biophysical Composition Index (BCI^46^) using the Tasseled Cap transformation coefficients from DeVries *et al*.^47^, the mean Normalized Difference BAreness Index (NDBAI^48^), the mean Enhanced Built-up and Bare land Index (EBBI^49^), the mean Normalized Difference Water Index (NDWI^50^), the mean Normalized Difference Built Index (NDBI^51^) and the Normalized Difference Urban Index (NDUI^52^). Synthetic aperture radar (SAR) imagery (2016–2018) is included as well, as this feature was previously found to be key^32,34,53^. In line with Demuzere *et al*.^32,34^, this study uses the Sentinel-1 VV single co-polarization backscatter filtered by the Interferometric Wide swath acquisition mode and both ascending and descending orbits, composited into a single image (hereafter referred to S1). From the S1 backscatter composite, an entropy and Geary’s C (a local measure of spatial association^54^) image is calculated, using a squared kernel of 5x5 pixels and a 9x9 spatial neighborhood kernel respectively. Finally, some other datasets are included such as the Global Forest Canopy Height product (GFCH^55^), the GTOPO30 digital terrain model (DTM) and derived slope and aspect from the U.S. Geological Survey’s Earth Resources Observation and Science (EROS) Center, the ALOS World 3D global digital surface model (DSM) dataset^56,57^ and a digital elevation model (DEM) by subtracting the DTM from the DSM. Note that the full set of features is processed on a resolution of 100 meters, following the default mapping resolution suggested by Bechtel *et al*.^31^. The reader is referred to Demuzere *et al*.^34^ for more details.

#### Training data

TA data are generally created by urban experts^31^, a time-demanding procedure, both because of the intrinsic nature of the task (i.e., the extent and heterogeneity of urban areas) and the ability of the urban expert to identify and digitize TAs consistently^58,59^. Here, expert TAs are used from nine U.S. cities: Phoenix and Las Vegas^60^, Salt Lake City^61^, Chicago and New York^62,63^, Houston, Washington D.C., Philadelphia and Los Angeles. The expert TAs from these cities are supplemented with polygons covering LCZ classes E (bare rock or paved) and F (bare soil or sand) to fully capture the spectral signature of the hot desert regions in the southwestern parts of CONUS.

A limitation of the expert TA procedure is that data are collected from only 9 cities (due to the time-demanding procedure described above). To address this limitation, additional training data are created based on a crowd-sourcing platform: MTurk (https://www.mturk.com). MTurk is highly scalable and allows for collecting a large number of urban and natural TAs across CONUS. The following process was used to collect MTurk TAs. First, MTurk participation is limited to workers with a Masters Qualification (i.e., users who have demonstrated high performance on MTurk in previous tasks) from English speaking countries (to avoid confusion from the instructions). The MTurk workers are shown a tutorial and asked to classify a satellite image (500 by 500 m) of an urban or natural area. For each satellite image (https://www.google.com/earth), users are also shown the corresponding Google Street View images (https://www.google.com/streetview/) within the 500 by 500 m area. Based on the satellite and Street View images, MTurk workers are asked to classify the area as a single LCZ. Locations are selected based on: (1) U.S. Environmental Protection Agency Air Quality Monitoring Sites (which are located in all major metropolitan areas) and (2) a supplement of manually chosen locations for LCZs that were under-sampled (from the 60 largest Urban Areas), to ensure that a wide range of built and natural environments are included. For each location, responses are obtained from at least ten unique MTurk workers; only when at least 70% of MTurk workers agreed on the classification (defined as the same LCZ or a near-neighbor LCZ), the MTurk TAs are included in the final training dataset.

Three different approaches (using TAs in the nine ‘expert’ cities) are used to compare the consistency between EX and MTurk TAs based on the degree of spatial overlap of the TA polygons: (1) full match where the MTurk TA falls completely within the EX TA, (2) match by centroid where the centroid of the MTurk TA is within the EX TA, and (3) match by intersection where the MTurk TA and EX TA intersect at some point in space. Using each approach we assessed to what degree the EX and MTurk TAs represent the same LCZ. The match percentage was 100% (n [number of matched polygons] = 8) for the full match approach, 87% (n = 69) for the match by centroid, and 65% (n = 141) for the match by intersection (Supplementary Table S1). While differences occur, the degree of consistency is actually higher compared to the results of HUMINEX (HUMan INfluence EXperiment^58,59^), that indicated large discrepancies between training area sets from multiple ‘experts’, nevertheless leading to strong improvements in overall accuracy when used all together. Combining expert and crowd-sourced data are therefore a reasonable approach to diversify training data for developing LCZ classification models.

**Fig. 2:**
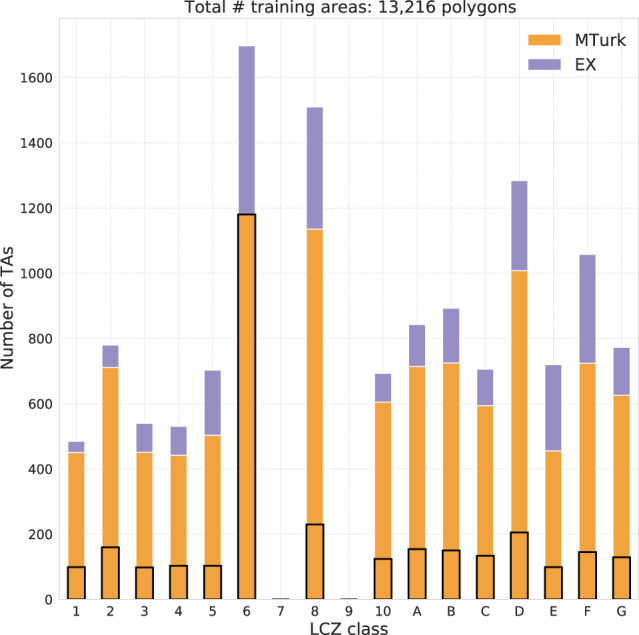
Number of expert (EX) and Amazon Mechanical Turk (MTurk) training areas used in the CONUS LCZ classification. Black boxes refer to the amount of original imbalanced MTurk TAs.

As a final TA preprocessing step, the surface area of large polygons is reduced to a radius of approximately 300 m, following Demuzere *et al*.^32,34^. These large polygons typically represent homogeneous areas such as water bodies and forests^58,59^, a characteristic that is neither needed nor wanted, as this leads to more imbalanced training data and computational inefficiency of the classifier. In addition, because of the imbalance of the MTurk TA sample (Fig. 2), the amount of all non-LCZ 6 MTurk classes (open lowrise) is increased five-fold, by randomly sampling five small polygons (100 x 100 m) from each original MTurk polygon (black boxes in Fig. 2), excluding LCZ 6 polygons. This results in a more balanced training set consisting of 13,216 polygons (10,323 MTurk and 2,893 EX TAs, Fig. 2).

### Classification procedure, quality assessment, and post-processing

As a final step in WUDAPT’s LCZ classification protocol, the random forest algorithm^64^ is applied, using the earth observation data and the labeled TAs^30,31^ as inputs. The accuracy of the resulting maps is then assessed in two ways, via (1) a pixel-based ‘random-sampling’ and (2) a polygon-based ‘city hold out’ procedure.

The random-sampling procedure is based on the default automated WUDAPT cross-validation procedure outlined in Bechtel *et al*.^45^ and is performed as described in Demuzere *et al*.^34^. Ten pixels are randomly sampled from each TA (resulting in a total of 132,160 pixels). From the resulting TA pixel pool, 50% is selected as the training set and the other 50% as test set based on a stratified (LCZ type) random sampling. This exercise is then repeated 25 times allowing us to provide confidence intervals around the accuracy metrics described in more detail below.

This strategy might lead to a biased accuracy assessment because of the potential spatial correlation in the train and test samples. Therefore, a second approach is applied in line with the methodology used in Demuzere *et al*.^32^. In this polygon-based city hold out procedure, TAs from all-but-one cities are used to train the classifier, while the remaining TAs from the held out city are used for the accuracy assessment. This is then repeated for all nine expert TA cities. As the information for training is independent of that used for testing, no bootstrapping is performed. The variability around the accuracy is, in this case, provided by the variable mapping quality for the different target cities. This city hold out approach is equivalent to cross-learning or model-transferability experiments in other recent studies^62,65–67^.

For both quality assessment approaches, the following accuracy measures are used: overall accuracy (OA), overall accuracy for the urban LCZ classes only (OA_*u*_), overall accuracy of the built versus natural LCZ classes only (OA_*bu*_), a weighted accuracy (OA_*w*_), and the class-wise metric F1^32,34,58,68–70^. The overall accuracy denotes the percentage of correctly classified pixels. OA_*u*_ reflects the percentage of classified pixels from the urban LCZ classes only, and OA_*bu*_ is the overall accuracy of the built versus natural LCZ classes only, ignoring their internal differentiation. The weighted accuracy (OA_*w*_) is obtained by applying weights to the confusion matrix and accounts for the (dis)similarity between LCZ types^58,70^. For example, LCZ 1 is most similar to the other compact urban types (LCZs 2 and 3), leaving these pairs with higher weights compared to e.g., an urban and natural LCZ class pair. This results in penalizing confusion between dissimilar types more than confusion between similar classes. Finally, the class-wise accuracy is evaluated using the F1 metric, which is a harmonic mean of the user’s and producer’s accuracy^68,69^.

According to Bechtel *et al*.^31^, the ideal scale for classification differs from the scale defined by the LCZ concept. More specifically, the optimal resolution for a pixel-based classification should be systematically higher than the preferred LCZ scale (hundreds of meters to kilometres)^27^, to account for non-regular and rectangular shapes of the patches. Consequently, single pixels do not constitute an LCZ and have to be removed. In the classical WUDAPT workflow, the granularity is reduced by a majority post-classification filter with a default radius of 300 m. This however has several shortcomings. Firstly, it does not account for distance, i.e. the center pixel is weighted as important as a pixel at the border of the filter mask. Secondly, it does not account for differences in the typical patch size between classes and consequently, linear features like rivers tend to be removed. Finally, it produces some artifacts. Therefore, a different filtering approach was chosen here. For each class the likelihood was defined by convolution of the binary membership mask derived from the initial map (1 if pixel is assigned to class *i*, 0 otherwise) with a Gaussian kernel with standard deviation *σ*_*i*_ and kernel size > = 2 *σ*_*i*_, resulting in a likelihood map per class. Subsequently the class with the highest likelihood was chosen for each pixel. Since the typical patches differ in size between LCZs, *σ*_*i*_ values of 100 m for LCZ 1, 250 m for LCZ 8 and 10 and 150 m for all remaining urban classes were chosen. For the natural classes, 25 m was chosen for water and 75 m for all other classes. Since these numbers were derived by experts, they introduce *a priori* knowledge to the procedure and deserve further investigation and adjustment in future. In particular, optimal *σ*_*i*_ are assumed to differ between cities and continents.

### Urban canopy parameter and population data

The LCZ scheme is considered to be a universal classification, that not only provides a common platform for knowledge exchange and a pathway to model applications in cities with little data infrastructure, but also provides a numerical description of urban canopy parameters (UCPs) that are key in urban ecosystem processes^71^. These UCPs, among others, include the building footprints (BF), average building height (BH), impervious surface area (ISA), the sky view factor (SVF), and the anthropogenic heat flux (AHF). Class-specific, globally generic, UCP ranges are provided in Table 1, and are especially useful in areas where such information is not available/incomplete and/or available at poor spatial/temporal resolutions^30,34^. CONUS does have such datasets available, which allows us to 1) evaluate the LCZ map with these independent datasets and 2) potentially fine-tune the existing generic UCP ranges provided by Stewart and Oke^27^. As outlined above, the LCZ typology is chiefly a description of land-cover but some of the types can be linked to land use and population. For example, compact highrise (LCZ 1) and midrise (LCZ 2) are generally associated with downtown commercial districts in most US cities, although it also includes tall residential apartment blocks. Compact lowrise (LCZ 3) are typically associated with densely occupied neighborhoods close to city centers, many of which were built in the early-twentieth century. Open types of all heights (LCZ 4–6) can be linked to the suburban residential areas. Finally, the large lowrise (LCZ 8) and heavy industry(LCZ 10) types are associated with storage units and large emitting facilities, respectively. In other words, one can expect each type to be associated with different populations. To evaluate the LCZs on the basis of this proposition, LCZ types are benchmarked against resident population counts as well.

#### Building footprints

The Bing Maps team at Microsoft released a nation-wide vector building dataset in 2018^72^. This dataset is generated from aerial images available to Bing Maps using deep learning methods for object classification. This dataset includes over 125 million building footprint polygons in all 50 U.S. States in GeoJSON vector format. The dataset is distributed separately for each state and has a 99.3% precision and 93.5% pixel recall accuracy. Since vector layers are highly challenging for large-scale analysis, Heris *et al*.^73,74^ converted the dataset to a raster format with 30 m spatial resolution in line with the National Land-Cover Dataset (NLCD) grid^29^, providing six building footprint summary variables for each cell. Our study uses the total footprint coverage per grid cell, with values ranging from 0 m^2^ (0%, no buildings) to 900 m^2^ (100%, completely built).

#### Building height

To our knowledge, there is currently no publicly-available, recent and high-quality building height (BH, *m*) dataset that spans the continental United States. Therefor, building height is taken from Falcone^75^, who provides a categorical mapping of estimated mean building heights, by census block group for the continental United States. The data were derived from the NASA Shuttle Radar Topography Mission, which collected ‘first return’ (top of canopy and buildings) radar data at 30-m resolution in February 2000. Non-urban features were filtered out, so that height values refer to object heights where urban development is present, e.g., buildings and other man-made structures (stadiums, towers, monuments). Due to difficulties in mapping exact building heights, information was aggregated on 216,291 census block groups across CONUS. In turn, block height values were categorized into six groups according to their statistical distribution and were categorized as ‘Low’, ‘Low-Medium’, ‘Medium’, ‘Medium-High’, ‘High’, and ‘Very High’. Using the building heights and footprints for 85,166 buildings in San Francisco (representative for 2010), the data quality was assessed (correlation of 0.8), and the mean and standard deviation (SD) of actual heights were calculated for block groups where actual building height data were available. This procedure resulted in the following mean (SD) height values: ‘Low’: too few observations to be meaningful, ‘Low-Medium’: 11.5 m (3.2 m), ‘Medium’: 13.1 m (3.1 m), ‘Medium-High’: 16.3 m (4.4 m), ‘High’: 21.7 m (8.2 m), and ‘Very High’: 35.3 m (14.2 m).

The procedure described above makes it clear that this dataset serves as a first-order proxy for actual building height data. Data were taken in the year 2000, which does not correspond to the year 2017 representative for this CONUS LCZ map. As such, benchmarking the LCZ map against this building height dataset neglects a net 6.7% increase in developed urban land, derived as the difference between the NLCD 2016 and 2001 developed land-cover classes^76^. Also, Falcone’s^75^ building heights are categorical and reflect the observed variability in San Francisco, which is not necessarily representative for all other CONUS urban areas. The spatial footprint is defined by census block groups, which vary in shape and scale as their original goal is to sample the population. The impact of these limitations is assessed using more recent, high-resolution and freely available datasets from the metropolitan areas of Austin, Boston, Des Moines, Los Angeles and New York, covering over 5 million buildings (Supplementary Table S2).

#### Impervious surface area

Impervious surface is taken from the National Land-Cover Database (NLCD) 2016 product^29,77^, which provides the percent of each 30 m pixel covered by developed impervious surface (range 0 to 100%). These authors created the dataset in four steps: (1) training data development using nighttime light products, (2) impervious surface modeling using regression tree models and Landsat imagery, (3) comparison of initial model outputs to remove false estimates due to high reflectance from non-urban areas and to retain 2011 impervious values unchanged from 2011 to 2016, and (4) final editing and product generation (see Section 6.1 in Yang *et al*.^29^ for more details).

#### Sky view factor

Information on sky view factor (SVF) is available for 22 U.S. cities (Atlanta, Baltimore, Boston, Buffalo, Cleveland, Denver, El Paso, Fresno, Las Vegas, Miami, Orlando, Philadelphia, Portland, Richmond, Salt Lake City, San Diego, San Francisco, San Jose, Seattle, Tampa, Tuscon, and Washington D.C.) and are obtained from Google Street View (GSV) images that are examined using a deep learning approach^78,79^. A complete sample of GSV locations in each city is retrieved through the Google Maps API; for all locations, an image cube is downloaded in the form of six 90-degree field-of-view images that face upwards, downwards, north, east, south, and west. The images are segmented by a convolutional neural network that was fine-tuned with GSV 90-degree images from cities around the world to yield six classes: sky, trees, buildings, impervious and pervious surfaces, and non-permanent objects^79^. Here, only the SVF is used, which is obtained by projecting the segmented upper half of the image cube into a hemispherical fish-eye to calculate the SVF using sky and non-sky pixels^80^. GSV images are inherently biased towards street locations, and thus greatly under-sample open spaces, including parks, golf courses, backyards, and natural areas in general^78^. Benchmarking with SVF data (ranges between 0–100%) is, therefore, only done for the urban LCZ classes within the CONUS domain.

#### Anthropogenic heat flux

Annual mean anthropogenic heat flux (AHF, *Wm*^−2^) data are provided by Dong *et al*.^81^, which are available globally at a spatial resolution of 30 arc-seconds. This product includes four heating components: energy loss, heating from the commercial, residential, and transportation sectors, heating from the industrial and agricultural sectors, and heating from human metabolism.

#### Population

Resident population counts representative for 2015 are provided by the Global Human Settlement global population grid (GHS-POP)^82,83^. These data are disaggregated from CIESIN’s GPWv4^84^ census or administrative units to grid cells with a resolution of 250 m, a manipulation that is informed by the distribution and density of built-up as mapped in the Global Human Settlement Layer dataset^3,5,83^. For other global and continental population datasets, and their fitness-for-use, the reader is referred to Leyk *et al*.^85^.

## Technical Validation

### Accuracy assessment

**Fig. 3:**
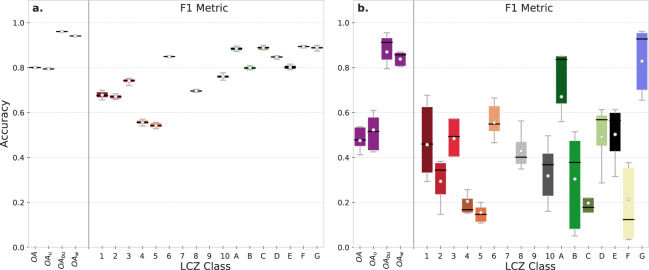
Overall and class-wise accuracies for the (**a**) random sampling and (**b**) city hold out approach. Colored boxes and grey whiskers span the 25–75 and 5–95 percentiles respectively. The means and medians are indicated by the white dots and black lines respectively. Note that the underlying confusion matrices are available in Supplementary Tables S3 and S4.

Accuracy assessment for the random sampling procedure using 132,160 pixels and all 41 earth observation input features result in scores of ≥80% for all OA metrics (Fig. 3a) (full confusion matrices are available in Supplementary Tables S3 and S4). The class-wise F1 metric shows larger variability with values for the urban classes between 55% (LCZ 5 - Open midrise) and 85% (LCZ 6 - Open lowrise), and >80% for all natural classes. The lowest accuracy is obtained for LCZs 4 (Open highrise) and 5, similar to previous studies^34^, and in line with the results of the 2017 IEEE GRSS Data Fusion Contest^62,86^ or Qiu *et al*.^67^ who found that LCZs 4 and 5 were difficult to distinguish. These LCZ types are characterised by the same building footprint and impervious surface areas, yet differ mainly due to the height of their roughness elements (≥25 m and 10–25 m respectively (see urban canopy parameter ranges in Table 1). This points to a current limitation of the input feature space^87^, i.e., apart from the S1 backscatter information, which responds to vertical elements in the landscape^88^ and the DEM information (which is subject to errors^56,57^), there is currently no publicly-available high-quality building height dataset spanning CONUS providing the much needed building height information to improve model performance (see also the ‘Urban canopy parameter data’ section).

Figure 3b shows that model accuracy is lower for the more stringent city hold out assessment, with average overall accuracy values ranging between 48% (OA) and 87% (OA_*bu*_). The average urban F1 metric ranges from 15% for LCZ 5 to 55% for LCZ 6, while average F1 values for natural classes range from 20% for LCZ C (bush, scrub) to 82% for LCZ G (water). These accuracies are only low to moderate. However, they can be considered as lower estimates of the real accuracy of the product, since most large urban agglomerations have specific training data and thus presumably higher accuracies. In addition, one has to keep in mind that much confusion occurs between similar classes, show-cased by the high weighted accuracy (*OA*_*w*_), ranging between 75 and 88%. Finally, our accuracies are within the range of accuracies reported from other transferability experiments using random forest classifiers^32,65^, acknowledging the somewhat higher accuracies using (residual) convolutional neural networks^65–67^. While the latter method is considered to constitute a feasible approach for automated large-scale LCZ mapping^66^, this feasibility to date has not yet been demonstrated.

### LCZ map and its relevance

**Fig. 4:**
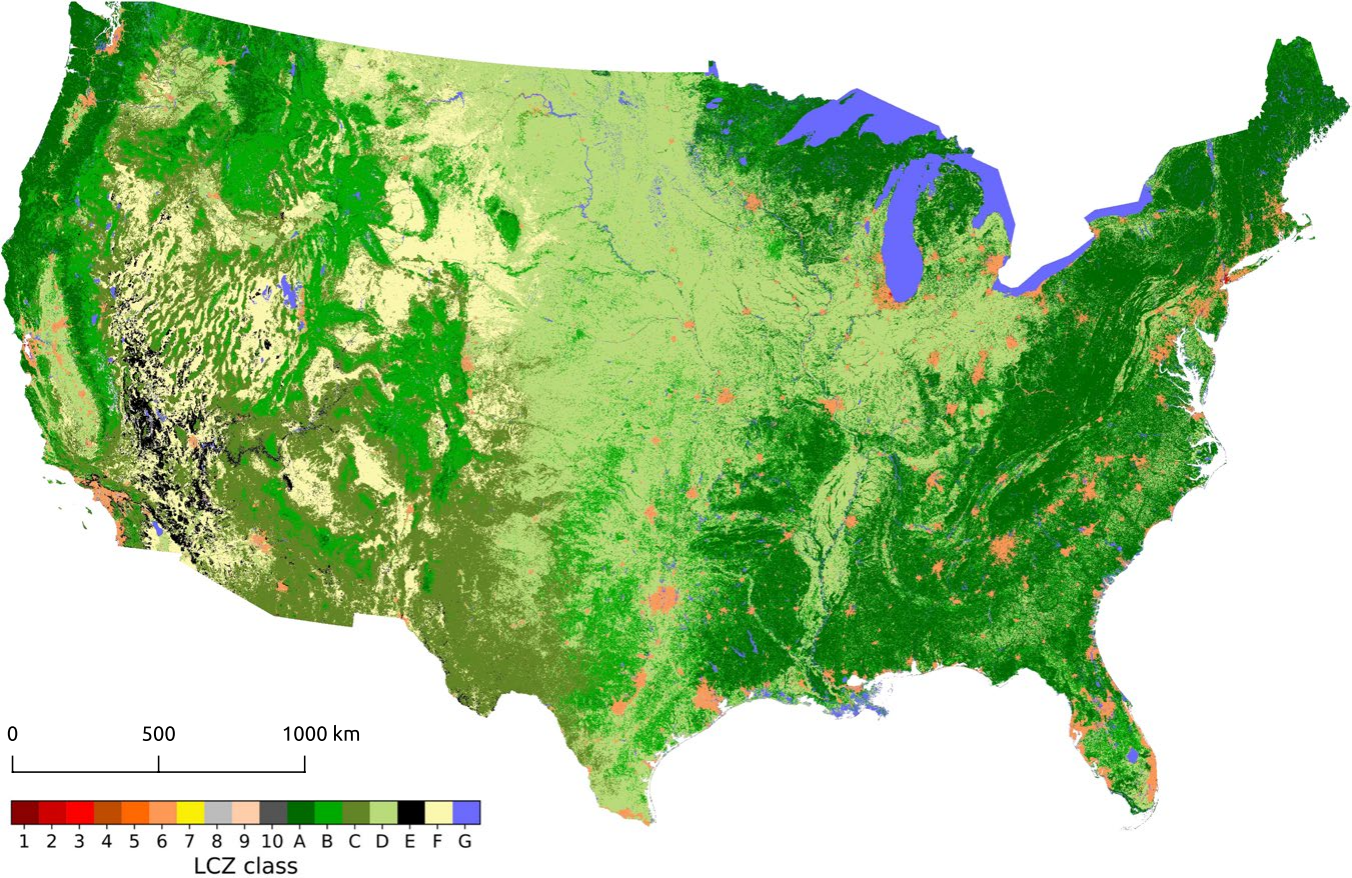
CONUS Local Climate Zone Map.

**Fig. 5:**
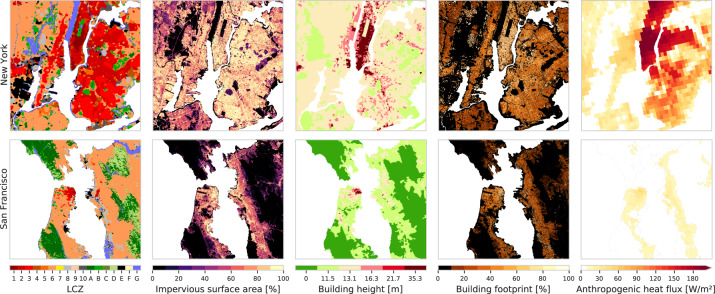
LCZ and urban canopy parameter maps for two selected urban areas: New York (top row) and San Francisco (bottom row). Examples of SVF maps for selected U.S. cities are provided in Middel *et al*.^78,79^. GHS-POP and other global population grids can be explored interactively via the POPGRID mapping tool ^85^.

**Fig. 6:**
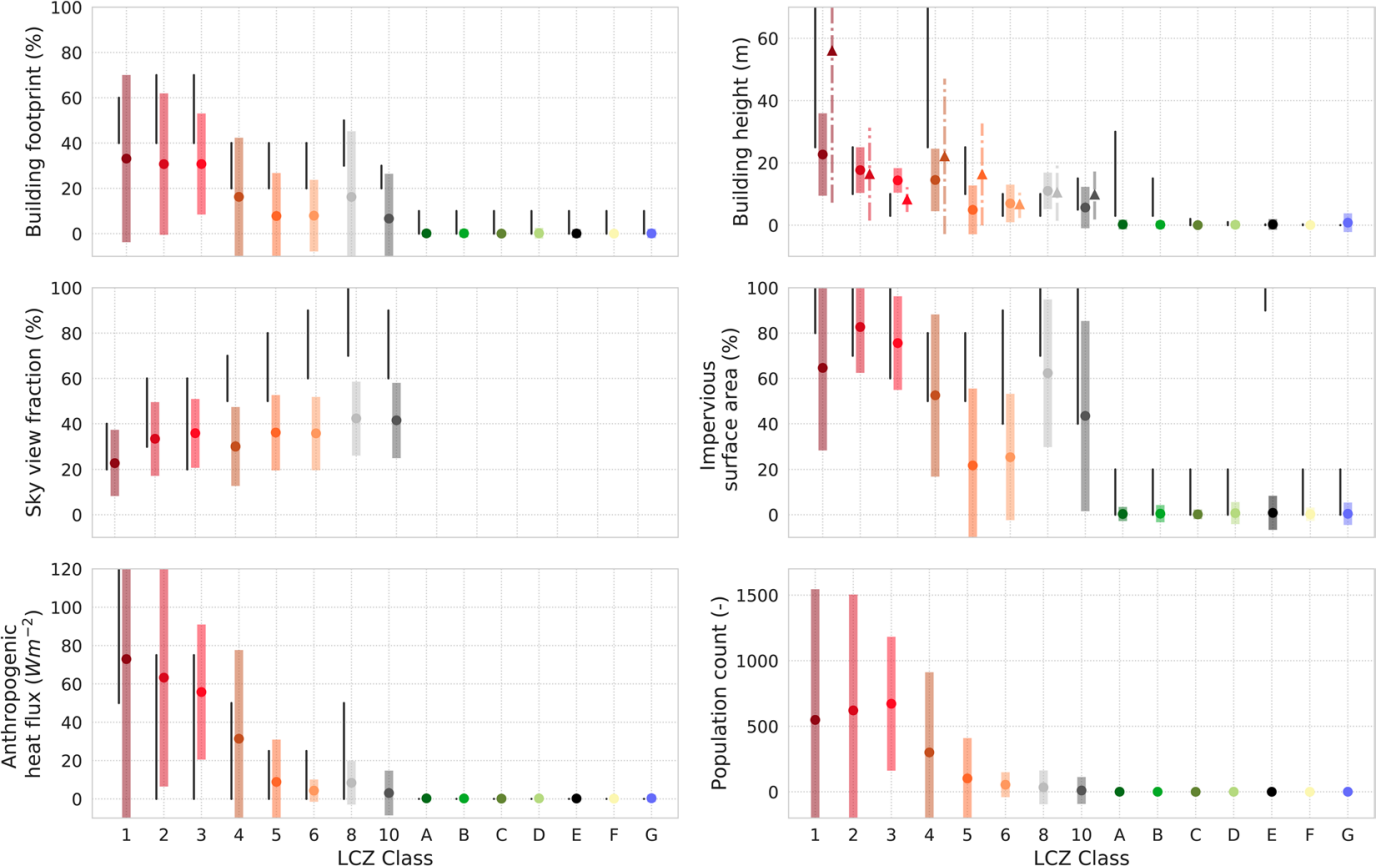
CONUS-wide urban canopy parameter ranges provided by Stewart and Oke^27^ (black lines, see also Table 1) and as a result from the spatial intersection between the final CONUS LCZ map (Fig. 4) and the urban canopy parameter and population datasets (colored bars) (Fig. 5). The colored dots present the mean, the extent of the bars ±1*σ*. Results from the metropolitan (five cities) building heights datasets (urban LCZ classes only) are depicted with triangles (mean) and dash-dotted bars (±1*σ*). SVF values are only benchmarked over the 22 cities for which such data are available, and are omitted for the natural LCZ classes. No reference population ranges are provided by Stewart and Oke^27^.

The resulting final CONUS LCZ classification, based on all TAs (13,216), all input features (41), and the random forest classifier in Google’s Earth Engine is shown in Fig. 4. The map is filtered using the morphological Gaussian filter described under ‘Classification procedure and quality assessment’, has a 100 m resolution, and is projected using the Albers Conical Equal Area NAD83 (North American Datum 1983) projection and NLCD grid. This map is used to benchmark the UCP data described in the following section and shown in Figs. 5 and 6.

Urbanization is an intrinsic part of the Anthropocene epoch (the human-influenced geologic time period^89^) in which urban areas represent a critical spatial nexus where climate drivers are concentrated, climate hazards are accentuated, and population exposure is greatest^90^. The effect of urbanization on the environment is an outcome of its physical form (i.e., the land-cover, the materials, and the geometry of buildings) and its functions (the transportation, energy usage, and generation of waste products) that sustain human activities. As these features and functions vary in space and time - and adversely affect local climate, hydrology, biodiversity, and air quality - it is crucial to characterize these urban properties to allow the assessment of their impact on human and environmental systems^1,30,91^. This CONUS-wide LCZ map contributes to this effort, and should be considered as a complementary source of information to existing and commonly-used land-cover maps such as the National land-cover Database^29^, which provides a limited number of urban classes (open space and developed low-, medium- and high-intensity areas). The latter classes typically reflect a degree of imperviousness, yet lack additional information on other types of urban characteristics that are key for various climate, weather, environment, and urban planning purposes^30,34,92^.

To this end, the CONUS LCZ map is benchmarked against the auxiliary urban form and population data described in section ‘Urban canopy parameter and population data’. As an illustration, Fig. 5 shows the LCZ map for parts of the New York and San Francisco urban areas, in conjunction with their benchmark data for impervious surface area, building height from Falcone^75^, building footprint, and anthropogenic heat flux.

Figure 6 provides a more in-depth analysis of the UCPs, comparing the theoretical ranges provided by Stewart and Oke^27^ with the CONUS-wide ranges estimated from the LCZ map presented here. For the SVF and lidar-based building height data (dash-dotted bars and triangles), this analysis is limited to the respectively 22 and 5 U.S. cities where these datasets are available. As LCZs are mainly related to structural and land-cover characteristics, no reference population ranges are provided by Stewart and Oke^27^.

Overall, UCP ranges match very well, yet differences occur which might be related to the confusion between LCZs 3, 4 and 5, and the characteristics and parameter representation of the underlying UCP data sources. LCZ-derived building heights are lower for LCZs 1, 4 and 5, and higher for LCZ 3. The benchmark against the more recent high-resolution lidar-based building height data (dash-dotted bars and triangles) is more in line with the ranges provided by Stewart and Oke^27^. This is most clear for LCZ 1, yet in general this behavior can be explained by the fact that 1) the metropolitan building height datasets are more representative in both time (more recent) and space (providing the actual building height for individual buildings, without categorizing and spatial averaging) and 2) NLCD classes 23 (developed - medium intensity) and 24 (developed - high intensity) show the greatest increase among the developed categories^76^. Also, the Stewart and Oke^27^ height ranges for LCZ A (dense trees) and B (scattered trees) are not visible in the benchmark against the Falcone^75^ nor the metropolitan products, as the former includes the height of all roughness elements (including trees), while the latter two are only representative for man-made structures. For the SVF, LCZ-derived values for LCZ classes 4, 5, 6, 8 (large lowrise) and 10 (heavy industry) are systematically lower than the range provided by Stewart and Oke^27^. These differences can be attributed partly to the difference in how SVF is obtained as part of the LCZ scheme versus that obtained by Google street view imagery. As the latter methodology is based on a perspective from the road surface, the derived SVF values generally do not account for urban spaces that are inaccessible to cars, such as backyards, parks, etc.^78,79^. This is also the motivation for omitting the natural classes from this analysis (Fig. 6). Finally, notwithstanding the various assumptions that have been made in the creation of both the LCZ map and the GHS-POP grid, the results are generally in line with expectations: the mean population counts in urban areas (LCZs 1–10) differ by type, and although there is considerable overlap, the compact types associated with higher built fractions have higher population densities than the open types. Concurrently, natural classes are characterised by very low population counts. As a first-order evaluation of the LCZ types, the population data are in agreement with the landscape categorization.

To summarize, this work represents the first CONUS-wide LCZ map - a data product that integrates building morphology and natural elements into existing datasets that describe land-cover. This result builds on previous city-based and continental-scale LCZ mapping efforts^34^, and adds to the growing globally consistent description of urban form and functions relevant to climate, weather, and the environment - the key mission of the WUDAPT project^30^.

The primary value of the LCZ scheme is that it can generate the urban data needed by climate models to simulate the impact of landscape change on the overlying atmosphere. As such, it is an integrative description of the urbanized landscape that accounts for the variety of urban characteristics that have climatic impacts. The continental scale map of LCZ types generated here can be used to evaluate the exposure of the urbanized landscape and population to hazards associated with current and future climate changes. These include for example the impacts of heat stress in the different types of LCZ and on those that reside and/or work there^93^. While cities are known to generate localized warming (urban heat islands), the impact of global climate change is expected to increase the frequency and magnitude of heatwaves, enhancing existing heat stress and risk in cities^93–95^. Finally, this work also demonstrates the possibility of integrating expert-derived and crowd-sourced training data in the LCZ mapping process. We expect that this data product will be useful to other researchers and practitioners who need descriptions of urban form and functions at the national scale to assess the impact of cities and urban planning on human and environmental systems.

## Data Records

The CONUS LCZ map provides a CONUS-wide LCZ classification for 2017 and is provided on a 100 m spatial resolution in the Albers Conic Equal Area projection (matching the projection of the NLCD maps^29^). All original training areas are combined in one shapefile, on the same NLCD projection. Both datasets are available via figshare^96^. The various urban canopy parameter datasets are freely available and can be obtained via their corresponding authors. All earth observation input features derived from Landsat 8, Sentinel-1 and some other data sources are freely available, and are computed on and stored as assets in Google Earth Engine.

## Usage Notes

Local Climate Zones were originally designed as a new framework for urban heat island studies, and therefore contain ‘natural’ land-cover classes that can be used as ‘control’ or ‘natural reference’ areas^27^. Yet the very few natural classes in the LCZ scheme can not capture the world’s existing natural variability, and can thus - with respect to the natural land-cover classes - not compete with other products such as the 16 natural land-cover classes from NLCD^29^ or the 20 and 36 layers that describe the Earth’s terrestrial surface in the Copernicus Global Land-Cover Layers^97^ and the European Space Agency Climate Change Initiative land-cover^98^ products respectively. In contrast, the strongest added value of the LCZ framework (and map) is the high number of urban classes, that are easily interpretable, globally consistent, and shown to exhibit class-specific thermal characteristics^87,93,99–107^. As this provides strong added value to other, often binary, global urban products (e.g. the Global Human Settlement Layer (GHSL^3,5^), the Global Urban Footprint (GUF^4^), the global annual urban database (GAUD^108^) and the Global Artificial Impervious Areas (GAIA^6^)), we advise users to combine the urban LCZ classes with any other land-cover product that provides a wider range of natural land-cover classes.

## Supplementary information

Supplementary Information

## Data Availability

All Google Earth Engine codes to pre-process the earth observation input features and perform the actual LCZ classification are available upon request. The pixel-based random-sampling assessment was done using the randomForest v4.16-14 package available in R-project^109^. The CONUS LCZ map figure is produced with QGIS v3.4. All other data processing and visualizations are done in Python v3.6.9.
