# Supplementary material for: Combining expert and crowd-sourced training data to map urban form and functions for the continental US
Source: Sci Data. 2020 Aug 11;7:264. doi: 10.1038/s41597-020-00605-z (PMC7421904; doi:10.1038/s41597-020-00605-z)
Supplement: Supplementary file 1 — Supplementary Information [file 41597_2020_605_MOESM1_ESM.pdf]

Supplementary Information for “Combining expert and crowd-sourced training data to map urban form and functions for the continental US”

Matthias Demuzere[1,*], Steve Hankey[2], Gerald Mills[3], Wenwen Zhang[2], Tianjun Lu[2], and Benjamin Bechtel[1]

[1]Department of Geography, Ruhr-University Bochum, Bochum, Germany
[2]School of Public and International Affairs, Virginia Polytechnic Institute and State University, Blacksburg, USA
[3]School of Geography, University College Dublin, Dublin, Ireland
[*]matthias.demuzere@rub.de

ABSTRACT

Although continental urban areas are relatively small, they are major drivers of environmental change at local, regional and global scales. Moreover, they are especially vulnerable to these changes owing to the concentration of population and their exposure to a range of hydro-meteorological hazards, emphasizing the need for spatially detailed information on urbanized landscapes. This data needs to be consistent in content and scale and provide a holistic description of urban layouts to address different user needs. Here, we map the continental United States into Local Climate Zone (LCZ) types at a 100 m spatial resolution using expert and crowd-sourced information. There are 10 urban LCZ types, each associated with a set of relevant variables such that the map represent a valuable database of urban properties. These data are benchmarked against continental-wide existing and novel geographic databases on urban form. We anticipate the dataset provided here will be useful for researchers and practitioners to assess how the configuration, size, and shape of cities impact the important human and environmental outcomes.

Table S1. Alignment comparison of the MTurk and EX TAs.

Match method by TA relationship	Match number of TAs	Match (%)
Full Match (MTurk is completely within the EX TAs)	8/8	100%
Match by centroid (The centroid of MTurk is within the EX TAs)	61/69	87%
Match by intersection (There are intersections between MTurk and EX TAs)	91/141	65%

Table S2. Metadata for building height datasets in US cities.

City	Creation	Updated	# Buildings	URL
Austin	Oct 31, 2017	Apr 10, 2020	454,686	https://data.austintexas.gov
Boston	Oct 21, 2013	Jan 7, 2014	129,370	http://boston.maps.arcgis.com
Des Moines	Jan 27, 2012	Apr 19, 2017	308,295	https://www.dsm.city
Los Angeles	2014	Nov 1, 2016	3,118,973	https://egis3.lacounty.gov
New York	May 3, 2016	Apr 6, 2020 (Weekly)	1,085,112	https://data.cityofnewyork.us

Table S3. Aggregated confusion matrix for the LCZ classification based on the random sampling approach, as the sum over all 25 bootstraps. UA and PA refer to User and Producer Accuracy respectively, expressed in %.

Classified as / LCZ Class	Ground Truth / LCZ Class: 1	2	3	4	5	6	8	10	11	12	13	14	15	16	17	UA (%)
**1**	***8979***	1822	7	929	550	353	394	426	3	0	0	0	7	0	5	**66.6**
**2**	1324	***17894***	449	884	1004	1875	1772	352	0	20	0	34	187	19	11	**69.3**
**3**	1	907	***13314***	30	173	5122	764	31	0	126	4	210	67	76	0	**63.9**
**4**	1847	2024	34	***9211***	1590	2317	2544	355	0	109	0	20	101	59	64	**45.4**
**5**	591	2419	221	1179	***15326***	9175	5319	428	53	155	13	244	200	342	35	**42.9**
**6**	8	444	630	185	658	***184039***	5261	577	593	537	196	1980	408	1071	538	**93.4**
**8**	51	935	150	182	978	15491	***50611***	1001	8	78	54	413	1461	640	172	**70.1**
**10**	174	534	64	102	199	2419	2353	***17165***	0	46	9	174	334	425	127	**71.2**
**11**	0	0	1	0	20	1418	1	17	***29103***	1686	121	559	3	90	56	**88.0**
**12**	4	30	63	54	70	2737	98	48	1996	***28852***	741	2399	66	469	298	**76.1**
**13**	0	0	0	0	1	892	88	66	301	562	***23910***	551	4	1469	6	**85.9**
**14**	0	67	93	17	144	4545	240	32	451	1540	247	***48508***	178	900	313	**84.7**
**15**	15	371	24	23	48	2436	2181	369	23	97	122	312	***20697***	434	48	**76.1**
**16**	9	15	78	8	19	2057	1093	82	32	123	546	1404	335	***51885***	239	**89.6**
**17**	0	11	0	39	60	1446	293	24	210	360	26	511	350	386	***22359***	**85.7**
**PA (%)**	**69.1**	**65.1**	**88.0**	**71.7**	**73.5**	**77.9**	**69.3**	**81.8**	**88.8**	**84.1**	**92.0**	**84.6**	**84.8**	**89.1**	**92.1**	

Table S4. Same as Table S3, but for the city hold out approach.

Ground Truth / LCZ class (columns); Classified as / LCZ Class (rows)

	**1**	**2**	**3**	**4**	**5**	**6**	**8**	**10**	**11**	**12**	**13**	**14**	**15**	**16**	**17**	**UA (%)**
**1**	***509***	107	0	131	44	77	23	91	0	0	0	0	1	0	0	**51.8**
**2**	204	***659***	79	172	120	280	183	92	0	0	0	0	13	0	0	**36.6**
**3**	1	296	***599***	27	69	1033	105	32	0	3	0	0	0	0	0	**27.7**
**4**	141	200	48	***229***	161	407	433	74	0	2	0	0	5	1	5	**13.4**
**5**	70	199	53	153	***281***	1238	462	100	0	2	0	2	9	6	1	**10.9**
**6**	4	37	105	8	155	***7132***	371	19	27	42	3	45	46	28	7	**88.8**
**8**	5	110	20	39	95	1603	***2545***	187	2	5	2	13	152	29	10	**52.8**
**10**	46	174	0	42	26	140	445	***488***	0	0	9	0	84	23	12	**32.8**
**11**	0	0	0	0	0	385	0	0	***1348***	94	7	13	0	1	1	**72.9**
**12**	0	0	5	1	0	981	9	0	219	***602***	18	283	35	5	5	**27.8**
**13**	0	0	0	0	0	222	29	1	82	111	***169***	96	228	157	0	**15.4**
**14**	0	1	0	1	2	774	18	2	32	486	27	***1177***	23	29	10	**45.6**
**15**	28	80	10	19	12	949	404	84	0	18	181	7	***1485***	304	7	**41.4**
**16**	0	0	2	0	5	863	313	59	0	30	440	380	478	***670***	4	**20.7**
**17**	0	0	0	0	0	140	33	4	9	12	5	3	23	31	***1216***	**82.4**
**PA (%)**	**50.5**	**35.4**	**65.0**	**27.9**	**29.0**	**44.0**	**47.4**	**39.6**	**78.4**	**42.8**	**19.6**	**58.3**	**57.5**	**52.2**	**95.1**	
